# Ethics in humanitarian settings—relevance and consequences for dialysis and kidney care

**DOI:** 10.1093/ckj/sfae290

**Published:** 2024-09-27

**Authors:** Valerie A Luyckx, Wim Van Biesen, Jadranka Buturovic Ponikvar, Peter Heering, Ali Abu-Alfa, Ji Silberzweig, Monica Fontana, Serhan Tuglular, Mehmet Sukru Sever

**Affiliations:** Nephrology Department, University Children's Hospital, University of Zurich, Zurich, Switzerland; Department of Public and Global Health, Epidemiology, Biostatistics and Prevention Institute, University of Zurich, Zurich, Switzerland; Renal Division, Brigham and Women's Hospital, Harvard Medical School, Boston, MA, USA; Department of Paediatrics and Child Health, University of Cape Town, Cape Town, South Africa; Renal Division, Ghent University Hospital, Ghent, Belgium; Department of Nephrology, Division of Internal Medicine, University Medical Centre Ljubljana, Ljubljana, Slovenia; Faculty of Medicine, University of Ljubljana, Ljubljana, Slovenia; KfH-Nierenzentrum, Städtisches Klinikum Solingen, Solingen, Nordrhein-Westfalen, Germany; Faculty of Medicine, American University of Beirut, Nephrology, Beirut, Lebanon; Section of Nephrology, Department of Internal Medicine, Yale School of Medicine, New Haven, CT, USA; The Rogosin Institute, New York, USA; Weill Cornell Medical College, New York, USA; New York-Presbyterian Hospital/Weill Cornell and Lower Manhattan Hospitals, New York, USA; European Renal Association European Dialysis and Transplant Association, Parma, Emilia-Romagna, Italy; Department of Nephrology, School of Medicine, Marmara University, Istanbul, Turkey; Istanbul University, Istanbul School of Medicine, Department of Nephrology, Istanbul, Turkey

**Keywords:** dialysis, disaster nephrology, ethics principles, health systems resilience, humanitarian action

## Abstract

With the increasing frequency and severity of disasters and the increasing number of patients living with kidney disease, on dialysis and with transplants around the world, the need for kidney care in humanitarian settings is increasing. Almost all humanitarian emergencies pose a threat to kidney health because all treatments are highly susceptible to interruption, and interruption can be deadly. Providing support for people requiring dialysis in humanitarian settings can be complex and is associated with many trade-offs. The global kidney care community must become familiar with the ethics, principles and duties essential to meeting the overarching goals of ethical and effective disaster relief. Ethics principles and values must be considered on the individual, public health and global levels. The wellbeing of a single patient must be considered in the context of the competing needs of many others, and optimal treatment may not be possible due to resource constraints. Public health ethics principles, including considerations of triage and resource allocation, maximization of benefit and feasibility, often become directly relevant at the bedside. Individuals delivering humanitarian relief must be well trained, competent, respectful and professional, while involved organizations need to uphold the highest professional and ethical standards. There may be dissonance between ethical guidance and practical realities in humanitarian settings, which for inexperienced individuals may present significant challenges. Sustaining dialysis care in emergencies brings these issues starkly to the fore. Preparedness for dialysis in emergencies is an ethical imperative that mandates multisectoral stakeholder engagement and action, development of surge response plans, clinical and ethics guidance, and transparent priority setting. This manuscript outlines common ethics challenges and considerations that apply in all humanitarian actions, and illustrates their relevance to kidney care as a whole, using examples of how these may apply to dialysis and kidney disaster relief efforts in humanitarian settings.

## INTRODUCTION

Humanitarian emergencies comprise both natural and man-made disasters. Mass natural disasters often receive rapid global attention, whereas armed conflicts soon become chronic and regarded as the ‘new normal’ [1]. Almost all of these emergencies pose a threat to kidney health [2]. People with previously healthy kidneys may develop acute kidney injury (AKI), some of whom will require dialysis. People across the spectrum of chronic kidney disease (CKD) and with kidney transplants are highly vulnerable to disruptions in care through damage to infrastructure, interrupted medication supplies and displacement. People on maintenance dialysis are a unique group who may be apparently well and have few symptoms, and yet die within days if dialysis is interrupted. Sustaining dialysis is therefore an urgent challenge for the kidney community in emergencies, and is highly dependent on robustness of pre-emergency dialysis services and resilience of the health systems. In the past, dialysis needs in emergencies have often been considered too hard to meet, given the complex logistical considerations, so dialysis services were often interrupted and patients had to be evacuated or died [3, 4]. Prioritizing the urgent needs of the dialysis population is challenging in the face of many other competing healthcare needs, which might be less immediately life-threatening, but may affect many more persons and may be more easily addressed. The increasing number of patients receiving maintenance dialysis or living with CKD or kidney transplants around the world is making it harder for humanitarian responses to overlook this population, but ethics challenges arise with regard to the obligations of the many stakeholders (Table 1) [5].

**Table 1:** Ethics dilemmas arising in kidney care in humanitarian settings.

**Dilemma**
Are some disasters more important than others?
Is this impacted by:
- Cause of emergency/conflict?
- Ease of logistics?
- Varying degrees of global solidarity?
- Aid fatigue?
- Discrimination and impartiality?
- Political rhetoric
- Fear
Does the number of patients affected in a crisis impact decision to intervene?
Is there a justification to intervene in acute but not chronic emergencies?
Does the international community have an obligation to support health services previously provided by the health system (under Universal Health Coverage)?
- If yes, at what cost? For how long?
- Is there a limit?
- What about when services are only paid for out-of-pocket?
Does the number of people at risk of dying if dialysis/kidney care services are interrupted have moral weight?
- If yes, what are the equity obligations of international organizations towards people living with other life-threatening conditions?
Are ethical values modifiable in large scale emergencies?
Should dialysis and transplantation needs be prioritized at all?
- If yes, then what are the international obligations to other patients living on dialysis under war or disaster circumstances when services are paid for out-of-pocket and not covered by the health system?
- Does this differ for acute vs maintenance dialysis?
If supplies are very limited should one compromise on care for all, or deliver adequate care for a few?
What are the obligations of the dialysis and pharmaceutical industries in disasters?
Where would this chain of analogous responsibilities stop?
Do professional nephrology organizations have a duty to advocate and mobilize support for these patients?
If there is no international obligation to help such relatively small, but highly vulnerable population groups, who need highly expensive therapies, how can this be explained/justified to the patients?
Who should support the local clinicians to cope with their moral distress?

Relief organizations, ideally in conjunction with ministries of health, must determine how best to allocate resources in emergencies to meet as many needs as possible [6]. Prioritization, planning exercises and sensitization of all stakeholders should be part of pre-emergency planning such that mobilization of support can happen efficiently and effectively. Since a delay in dialysis may mean death, quick action is required. Support teams should be well informed in advance of the specific needs, considerations and available practical solutions for kidney care [2]. Many countries and health facilities lack emergency preparedness plans. Here we outline common ethics considerations that apply to humanitarian action which should include meeting the needs of all people living with kidney disease. We focus on examples of how these may apply to dialysis provision in humanitarian settings given that dialysis represents the most obvious tip of the iceberg of kidney care needs and is associated with many challenges. It is critically important however that while prioritizing dialysis needs, the needs of others with kidney disease are not overlooked.

## ETHICS CONCERNS IN HUMANITARIAN SETTINGS

Humanitarian action is complex and not without risk of physical, economic, social and moral harms, and therefore cannot be presumed to be only good and noble, and cannot be rushed into lightly [7].

### Ethics principles and values

As with any medical encounter, the basic principles of biomedical ethics—autonomy, avoiding harm, doing good, justice—as outlined by Beauchamp and Childress [8] apply also in humanitarian settings (Table 2). Provision of medical care in these settings differs from routine care in many ways, including that the clinician may not only be able to focus on the wellbeing of a single patient, but may need to consider the competing needs of many patients simultaneously, and may not always be able to assist every patient as they would feel necessary under usual circumstances. As such, public health ethics principles often become directly relevant at the bedside, which include considerations of triage and resource allocation, maximization of benefit and feasibility concerns (Table 2) [9]. There may also be dissonance between ethics guidance and the practical realities in humanitarian settings, all of which, especially for inexperienced individuals, may be very challenging to negotiate [10]. Application of public health ethics principles and values requires oversight from leadership. Ethics principles should guide healthcare decision makers—ministries of health, district health administrators and non-governmental organizations (NGOs)—and should provide clear guidance for clinicians regarding resource allocation and optimal care to shield them to some degree from the moral distress of making life and death decisions alone at the bedside [11–13].

**Table 2:** Relevant ethics principles and values to be followed during interventions in humanitarian settings.

Domain	Principle/value^a^	Relevance in humanitarian settings
Biomedical ethics	Autonomy	• Keep patients informed at all times
		• Accept that not all patient choices can be acted upon
	Avoiding harm	• Adhere to professional standards—quality of care
		• Distinguish between acute and chronic patients if limited dialysis resources
		• Be aware of resources available
		• Be aware of/manage moral distress among healthcare workers
		• Support locally led response
		• Avoid unnecessary procedures/interventions
	Caring	• Maintain the duty to care across spectrum of care, including palliative care
	Doing good	• Advocate for access to dialysis (and the spectrum of kidney care)
		• Optimize quality of dialysis
		• Adapt care delivery safely if needed
		• Prepare crisis clinical guidance/guidelines
	Justice	• Aim for equity
		• Develop/adhere to transparent triage principles
		• Maximize benefit
		• Include needs of the most vulnerable
	Respect for privacy and confidentiality	• Stewardship of clinical data
		• Collect research data
		• Monitor quality of care
Public health ethics	Accountability	• Support triage decisions
		• Justify resource allocation decisions
	Cost-efficiency	• Maximize benefit at lowest cost (without cutting unnecessary corners)
	Equal moral concern	• Consider needs of the most vulnerable
	Fair distribution of benefits and burdens	• Monitor impact of resource allocation decisions on equity
	Inclusiveness	• Avoid discrimination
	Political feasibility	• Engage with policy makers
	Proportionality	• Prioritize resource allocation to those who have been most harmed
	Protecting workers	• Ensure adequate training, resources, safe work environment, guidance/guidelines
	Reasonableness	• Include dialysis provision in disaster planning especially if previously provided under state/UHC and/or to a large number of patients
	Reciprocity	• Consider priority for instrumental value—e.g. prioritize health workers for interventions to keep them healthy
		• Support healthcare workers
	Responsiveness	• Plan ahead and ensure adequate surge capacity for dialysis
		• Plan for needs of patients with transplants, CKD
	Solidarity	• Develop intergovernmental arrangements for refugees requiring dialysis and kidney care
	Transparency	• Communicate clearly about justification for decisions taken, support accountability
	Trust	• Communicate consistently, non-abandonment fosters trust over time
	Utilitarianism	• Consider how to optimize action for greatest good
	Moral distress	• Support for healthcare workers who are overwhelmed with workload, personal challenges
		• Support healthcare workers needing to make life-and-death decisions at bedside
Humanitarian principles	Humanity	• Recognize humanity of all affected by disasters
	Impartiality	• Do not judge, support all who are in need
	Independence	• Avoid corruption, resist pressure to compromise on mission and goals
	Neutrality	• Do not take sides in a conflict
	Respecting culture and customs	• Be careful to work within the system, and support locals rather than dominate
	Solidarity	• Assist others as one would hope to be assisted

Derived from [8, 9, 14, 16, 83].

^a^Principles/values listed alphabetically, not in order of priority.

UHC, universal health coverage.

In addition to ethics principles, basic humanitarian principles including respect, solidarity and impartiality should be adhered to during any intervention (Table 2) [14]. Humanitarian settings are however not uniform. Disaster type and scale, location, duration and pre-disaster circumstances all impact the ethics challenges faced both on the ground and beyond. Different ethics principles may apply in a pandemic compared with a massive earthquake or a war, but the need to respect these principles under all circumstances is clear.

### Prioritization in humanitarian settings

#### Resource allocation

Supply and demand mismatches arise rapidly in humanitarian settings, especially with respect to highly resource-dependent activities such as dialysis [15–17]. If infrastructure is damaged or inaccessible patients may have no or reduced access to maintenance dialysis; if large numbers of patients experience crush syndrome and AKI, the demand for existing services will surge. In some settings, despite an acute need, if dialysis was not available before the crisis, it is practically challenging to deliver this soon enough to save many lives. Decisions must be made about whether or not to allocate (any) resources to support dialysis in such settings, as subsequent withdrawal of such services at the end of an intervention may create significant long-term harm.

Various principles have been outlined to guide resource allocation under conditions of scarcity (Table 3). Classically, the utilitarian principle, which entails allocation of resources to maximize overall benefit has been the most accepted. Utilitarianism may prioritize those with instrumental value, i.e. saving the lives of those who can be useful to society, e.g. healthcare workers, or may consider allocation of resources to those who would benefit most, which may prioritize younger over older salvageable individuals to maximize life-years saved [18–21].

**Table 3:** Potential dialysis allocation or rationing strategies.

Strategy	Benefit	Harm
Ability to pay (Libertarianism)	• Self-selection of candidates	• Highly inequitable, favours those with resources over others
	• Chance of sustainability	
Random chance (Egalitarianism)	• Impartial, all treated equally (lottery, luck)	• May not address the needs of the sickest, youngest, etc.
	• Physician and patients do not decide	• Cannot operate when no vacant dialysis slots are available
Treat first-come first-served (Egalitarianism)	• Physicians and patients do not decide	• May not address the needs of the sickest, youngest
		• Those with resources to get to a dialysis centre have an advantage (tend to be bread-winner men in LMIC)
Give priority to the sickest (Prioritarianism)	• ‘Rule of rescue’	• Requires accurate determination of relative prognosis
	• Prioritize those who need most	• Some may be too sick to benefit, present too late
		• May incur futile use of resources
Give priority to the youngest (Prioritarianism)	• Gives chance to those who have most life-years to gain	• Discriminates against older possibly more productive individuals
		• Young age on dialysis does not mean long survival, ideally need transplant available for maximal benefit
Maximize lives saved (Utilitarianism)	• Maximizes efficiency • Focus on treating the most ‘salvageable’—would favour treating AKI over KF	• Discriminates against those with comorbidities, other terminal illness
		• Discriminates against KF in favour of AKI
Treat those who would benefit most (Utilitarianism)	• Maximizes efficiency	• Triage cases and treat the ‘healthiest’
	• Focus on treating the most ‘salvageable’	• When a new patient would benefit more than an existing patient, creates a dilemma of whether to remove existing patient from dialysis if no vacant slots?
		• Prioritize AKI over KF for limited slots (e.g. in pandemic)
		• Determination of ‘salvageability’ may not be objective
Treat those who are more useful to society (Instrumental value, Utilitarianism)	• Efficiency beyond dialysis	• Problems in evaluation of ‘usefulness’
	• Favours breadwinners, parents, employed, i.e. ‘social worth’	• Discriminate against very young, elderly, those with comorbidities
Reciprocity (Personalism)	• Favours those who contributed in the past	• Discriminates against those who have not had a prior opportunity to contribute
Redressing injustices (Personalism)	• Minimize differences between population groups	• Discriminates against groups that historically may have done wrong, but present members (e.g. children) may not have participated in wrongdoing (e.g. apartheid)
‘Complete lives system’ (Prioritarianism)	• Favours adolescents and young adults	• Discriminates against very young and older
	• Focus on ‘lives’ rather than ‘experience’	• Discriminate against those with more comorbidities
	• Favours those with better prognosis	
	• Uses lottery to decide among equal candidates	
Non-abandonment (Egalitarianism)	• Tries to allocate limited resources fairly and proportionately across all high cost chronic diseases within a limited budget	• Not all individuals gain access to care
	• Rationing fairly	
Ensuring an acceptable minimum standard for all (Sufficientarianism)	• Improve health of all up to a minimum level	• Favours provision of primary care and prevention, early diagnosis treatment under UHC for kidney disease
		• Under resource limitations—unlikely to justify state provision of dialysis until the minimum ‘sufficient’ baseline is met for all first
		• Does not consider inequities above the minimum threshold to be unjust, e.g. provision based on ability to pay
		• May justify reducing ‘quality’ in order to increase numbers of patients that can be dialysed (e.g. providing HD twice instead of 3 times a week)

Table extrapolated from concepts summarized by Persad *et al*. [15] and others [84–90]. Reproduced from [91]; published in [6].

‘Complete lives system’: aims to promote equality in achievement of ‘complete lives’; Egalitarianism: all people are equal and should be treated equally; Libertarianism: strong emphasis on liberty; Personalism: values the person and social dimensions; Prioritarianism: gives extra weight to the worst off in the interests of equity; Sufficientarianism: ensure that all have enough to meet a minimum standard; Utilitarianism: aims to maximize overall benefit.

LIC/LMIC, low and low-middle income countries; KF, kidney failure; UHC, universal health coverage; HD, haemodialysis.

Implementation of strict utilitarian principles in humanitarian settings, where logistics for most medical care is challenging, would almost always exclude ‘spending’ resources to meet the needs of those who require dialysis. This however violates the ethics principle of justice, by exacerbating the underlying inequities already associated with kidney failure [7, 22]. Paradoxically, given that dialysis is immediately life-saving, the utilitarian principle of treating those who would benefit most could arguably be applied to dialysis. Other ethics principles and values also favour some resource allocation to vulnerable patient groups, which include not abandoning the sickest, thereby mitigating intrinsic disadvantage, and granting people who need dialysis equal moral value with others (Table 3) [16]. Different groups may therefore be prioritized following different value preferences. Crucially, whichever principles are chosen, it is imperative that transparent guidance is in place to ensure consistent application in each context.

#### Triage

Under crisis conditions, triage is mandatory. When implementing triage or rationing guidelines in emergencies, the ethics principle of autonomy (respecting patient choice) is often overridden by the principles guiding resource allocation (Table 3) [23]. As recently experienced during the pandemic, some national guidelines advocated that intensive care unit care not be initiated, or even be discontinued, in cases considered ‘futile’, to create space for more salvageable individuals [24]. In justifying these practices, many have argued that there is no real moral difference between withholding and withdrawing care [25]. These arguments may have weight when applied to individual cases, but when in an emergency a service is interrupted and effectively ‘withdrawn’ from a whole population because it deemed too complex, the moral value between withholding and withdrawing may well change. An example here is the current situation in Sudan [5]. Most patients on maintenance dialysis in Sudan were not imminently dying prior to the war; therefore, the slow global response that contributed to interruption of treatment across the country seems extremely harsh in an otherwise non-‘futile’ setting.

The practice of triage may be morally disturbing but it is often necessary to avoid waste of resources and an increase in overall suffering. As commented by Waeckerle [26] ‘the ultimate act of commitment after a disaster is to abandon our duty to one person in favor of stabilizing others… Medical personnel who cannot overcome the horrors of a catastrophe or alter their philosophy of care must recognize their limitations and remove themselves…’. As hard as this may seem, especially in mass disasters where resources are scarce, triage may be considered a moral duty, to maximize benefit and minimize harm overall. Care ethics should always apply however, and if a decision is taken against provision of dialysis or other care for a specific individual on a specific day, it is imperative that all possible efforts are made to ensure the patient's comfort and dignity [23].

### Duty to rescue

Taking a consequentialist view, if an organization has the capacity to rescue and would experience minimal or no risk, there is a duty to rescue, although the extent of this duty may vary [27, 28]. International missions do not rush in after every emergency, and indeed humanitarian action tends to follow large and high-profile disasters (Box 1) [29]. Humanitarian intervention must be guided by the principles of accountability, transparency, responsiveness and appropriateness, with mindful stewardship of resources (Table 2). Relief organizations do not have infinite resources and it is rare that emergencies happen in isolation, therefore inherent in the choice to rescue would be prioritization of one emergency or one service over another. At times countries may choose to rescue and/or provide support to others, e.g. provide vaccines, out of self-interest rather than true solidarity [30]. Conflict settings pose additional political challenges [31]. Human rights violations are common, patients and healthcare workers are not safe, neutrality may be challenging to maintain, health centres may become targets and the global community may not (be able to) intervene [32].

BOX 1: **Global inequities in dialysis disaster responses.**

Armed conflicts/wars differ from other disasters, since they often become protracted and delivery of aid may be physically challenging. Three ongoing armed conflicts, in Ukraine, Sudan and Gaza, are highly illustrative of the challenges associated with supporting dialysis services in humanitarian settings (Table 4). Whereas there are commonalities between the three wars, response was substantially different. For Ukraine, in contrast to the other two countries, there was immediate action from European Union and the world, and dialysis supplies were relatively rapidly and sustainably procured and delivered, compared with the latter two crises, where support has been slow (Table 4) [38, 71]. In both Sudan and Gaza, dialysis supplies have been intermittently received, but infrastructure has been severely damaged or inaccessible. Dialysis staff had to flee, many continue to work without being paid. Dialysis provision has been significantly restricted for individual patients (reduced time and frequency of HD) in order to cater to high numbers of patients and limited functioning dialysis machines and units. In Sudan, funds to purchase supplies have been very hard to access, donations have been minimal, and transportation of supplies challenging and very costly. In Gaza, donations and funds have permitted the purchase of supplies but these supplies have not easily been permitted to cross the border given restrictions on the number of trucks allowed to cross per day and the competing trucks with other necessary supplies.

**Table 4:** Comparison of global response to the three most recent conflicts.

	Ukraine	Sudan	Israel-Hamas (Gaza)
Patients on haemodialysis pre-war	8717	8000	1000
Patients on peritoneal dialysis pre-war	913	120	0
Transplanted patients pre-war	1533	4500	500
Cost of kidney replacement therapy pre-war (USD)^a^	107 443 382/year (2018)	24 000 000/year	16 000 000/year
Pre-war dialysis funding from	State/UHC	State/UHC	Donors
Support for procurement and shipping of supplies	Rapid procurement/global support	Slow donations/slow procurement	Rapid donations/slow delivery
		Stockouts × weeks	
Evacuation of patients	Approx 700 to EU/400 to Russia	Unknown	Very few (paediatric) patients
Consequences	Kidney care services strengthened by challenges, transplantation increased, strong support by neighbour countries	Many patients dying during repeated stockouts or because of no dialysis slots	Patients dying from lack of dialysis and injuries

Adapted from [5, 38, 92, 93].

^a^Costs extrapolated to annual costs from reported data.

USD, US dollars; UHC, universal health coverage; EU, European Union.

Many patients and dialysis staff in Ukraine were independently able to relocate either to safer areas within the country or to surrounding countries. The Ukranian kidney societies rapidly developed strategies to coordinate care, distribute supplies, share data on needs, patient and service locations, and have even scaled up transplantation since the start of the war [78]. Such coordination and mobilization of resources within the country have not been possible in Sudan or Gaza, given the relatively weak health systems, tenuous dialysis services pre-war, interruptions in communication and importantly, limited interest of the global community. In other chronic war zones, including the Democratic Republic of Congo and the Tigray region in Ethiopia, many patients died because of interruptions of dialysis as these regions did not receive external support [3, 79]. Support for dialysis in other long standing crises such as in Syria and Yemen is being provided on an *ad hoc* basis by various donors [54, 80, 81].

The duty to rescue is in part relational [33]. Accordingly, if a government, which is morally obligated to protect its citizens, has the capacity to rescue but chooses not to, external actors may have a lesser obligation to do so. However, if a marginalized and neglected population within a country requires assistance that is willingly being withheld by the government, the international community may have a responsibility to act [28]. Also, the nephrology community could be seen to have some ‘relational duties’ to mobilize support for their colleagues and patients under their care who are not high on the priority lists of most NGOs and governments, by raising awareness and advocacy, and support, if feasible, via telemedicine or facilitating evacuation [5, 27, 34, 35]. We as nephrologists advocate for dialysis and to avoid interruptions in access to medication and care for those with CKD and transplants; patients with other illnesses have equal moral value and others should advocate for their needs.

The perceived duty to provide rescue dialysis may be influenced by how and through whom services were financed and organized before the emergency. In Tigray for example, dialysis was only accessible with out-of-pocket payment. Despite attempts by local nephrologists to raise awareness of the plight of patients during the conflict, dialysis services were not supported by the global community and most patients died [3]. In North West Syria and in Gaza, external support from international agencies and donors provided basic services and these agencies and donors persist in trying to support dialysis during the crises [36, 37]. In Ukraine and Sudan, dialysis had been provided under universal health coverage before the conflicts began, however the West mobilized to support Ukraine but support for Sudan has been minimal [38]. In practice, once crises become chronic, there is a tendency for the sense of duty to rescue to diminish and for humanitarian assistance to wane [1].

Recent experiences raise questions as to the obligations of neighbouring countries, following the principles of solidarity and reciprocity, in terms of accepting evacuated patients with complex kidney care needs. Refugees needing dialysis from Ukraine were rapidly hosted in European countries, by decree from the European Union, many receiving similar care to the citizens of the host countries, including transplantation [39]. If surrounding countries themselves are struggling however, who has the obligation to support them [40]? Evacuation of patients from Sudan has been hampered by limited personal resources of patients and visa challenges, and from Gaza has been restricted politically. Factors underlying differential responses to similar populations in need in different countries may include implicit bias, media attention, politics and self-interest, which may be difficult to influence. The duty to care therefore may exist in all emergencies, but equitable fulfillment of this duty around the globe, and on whom this obligation falls requires multi-stakeholder deliberation and reckoning.

### Duty to care

In humanitarian settings clinicians must find a balance between the duty to care, and personal risk [41, 42]. Medical professionals possess the required knowledge and skills to do good. Therefore, if the risks to themselves are not excessive in an emergency, and the expected benefit to patients is reasonable, medical professionals—including physicians, nurses, technicians, laboratory personnel—have a duty to care [41]. The duties of care of healthcare workers may come into conflict with their need to protect themselves and their families, as was evident during COVID-19. These professionals also cannot be expected to work 24/7, without basic resources or under unsafe conditions [43]. As such the clinician's duty to care in turn imposes obligations on the health systems and governments to maximize their safety in emergencies [14]. Following the utilitarian principle of instrumental value, most countries indeed prioritized healthcare workers for vaccination during COVID-19 (Table 3) [44].

#### Duty of competence

Recognition of our shared humanity may drive people to volunteer to help, but this comes with important obligations and responsibilities [42, 45]. Obligations of relief organizations include being transparent, collaborative, responsive to the needs on the ground and well prepared, and ensuring their staff are clinically and culturally competent [46]. The quality of humanitarian responses depends on the skills, training and professionalism of the humanitarian actors, all key to maximize benefits and minimize harms. Clinicians must recognize their own capacity and their own limits [26, 42, 46, 47]. Especially for expatriate clinicians, despite there being less regulatory oversight in an emergency, being elsewhere does not justify attempting procedures or delivering care that one is not qualified and competent to deliver [48, 49]. Such actions should be regarded as malpractice in humanitarian settings as they would under other circumstances, as the Good Samaritan principle does not support irresponsible actions [50]. Unnecessary procedures (e.g. placement of arterio-venous fistulae in maintenance dialysis patients by rescue teams) may be inappropriate in emergencies and can lead to complications, e.g. infections, that may necessitate further use of limited resources. Stewardship of resources and avoiding harm are key ethics principles underlying the duty to care.

Expatriate clinicians must also respect humanitarian principles (Table 2), be cognizant of local socio-political contexts, remain impartial, be willing to work within structures of NGOs (and not arrive as solo rescuers), and respect and collaborate with local healthcare workers given the inherent asymmetries of power [14, 49]. Human skills, the ability to be flexible, deal with the unexpected, and communicate effectively within and between teams with high turnovers, are critical in rescue workers [47, 49].

Dialysis rescue workers must have knowledge and skills required to deliver safe and effective dialysis under restricted circumstances, and have the experience to troubleshoot and find safe solutions if resources are lacking. In line with the principles of subsidiarity and accountability, relief interventions should be integrated into local practice and delivered such that they support the local health system and staff and enhance resilience [14].

An important consideration for external actors supporting dialysis in a humanitarian setting is how and when to leave [51]. External relief organizations may provide free dialysis that may not have been available or accessible at baseline [52]. This may create new expectations within a community, which if not sustainable will lead to a sense of betrayal and future mistrust.

### Standards of care

**Figure 1:**
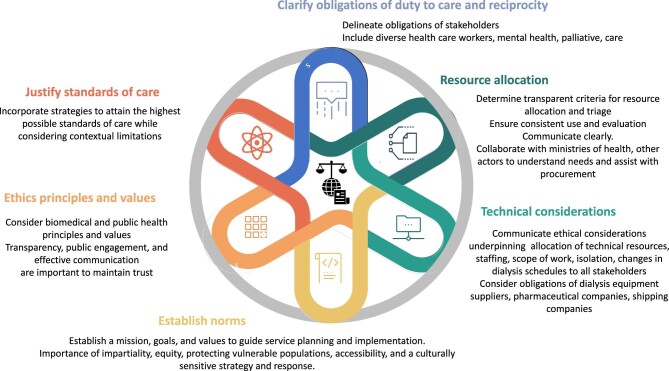
Ethics principles and considerations for planning and quality of kidney care. Ethics considerations apply to all components of planning and quality of care have ethical implications. Ethics frameworks must be agreed upon to strengthen health systems resilience in crises. Adapted from [82] under the terms of the Creative Commons CC-BY license.

Despite significant deviation from usual care in emergencies it is important to attempt to deliver quality care [14, 48, 53]. Consensus crisis standards of care should be developed before a disaster occurs, taking ethics principles and obligations into account, and must be disseminated and taught [2, 53, 54]. These should outline clear minimum standards for dialysis (Fig. 1). Compromises (e.g. reduced dialysis frequency) may be urgently required for a few days, but cannot be prolonged. There is a danger in protracted emergencies that the deviations from acceptable standards of care continue. It is important to avoid the slippery slope of normalizing such deviations or accepting unnecessary compromises, as over time patients will deteriorate and die [55]. Core indicators for quality of care should be developed for emergency contexts and monitored to ensure standards are acceptable [56]. The responsibilities of the various actors involved must be outlined. Decision making should be transparent and decision-makers held accountable for their decisions (Fig. 1) [53]. Clear communication is crucial to inform patients early about the limitations in care, to support their autonomy and capacity to protect their own health [46].

To maximize responsiveness, provision should be made to meet potential ancillary care needs. For example, dialysis may be a patient's only point of contact with the health system and they may have the expectation and trust that additional needs, such as management of diabetes or infections, will be addressed [45].

### Ethics in governance of disaster response

If health systems are resilient, healthcare workers are well trained and patients are well informed, countries may be well prepared and able to cope with many emergencies independently [2]. Conflict, civil wars and large scale and/or complex emergencies however often necessitate external assistance. Assistance can take multiple forms: humanitarian, military, financial or in-kind, and development aid [57]. Emergencies also often occur in fragile settings, where multiple vulnerabilities coexist. In these circumstances, ethics considerations extend beyond the traditional ethics principles that relate to triage and health resource allocation. The ethics considerations may also differ by the type of disaster. Overall, humanitarians must consider how to contribute the most benefit with the least risk of harm, and at times how to balance donor or NGO agendas and mandates with responsiveness to needs on the ground [57]. Transparency and oversight of external aid are imperative to reduce duplication of efforts in some areas and neglect of others. Donor agencies should be responsive to comprehensive needs on the ground, e.g. donation of funds purely for dialysis supplies but not for the required medications or running costs of a dialysis unit, including staff salaries, does not permit effective delivery of dialysis. Competing interests, hidden agendas, coercion and corruption may undermine humanitarian action [45]. Humanitarian actors must also be protected from harm [32]. Transparency and accountability are important principles to guard against these challenges and promote equity.

Obligations of governments and health decision-makers are clear, and resources are available to guide planning of all aspects of kidney care [2]. Many stakeholders in addition to governments are involved in the delivery of dialysis, therefore planning strategies must be inclusive, comprehensive and adapted to local contexts and capacity. For example, planning to initiate peritoneal dialysis where it has not been done before may not be feasible, and may cause significant harm and drain resources. The ethics principles of maximizing good and avoiding harm should apply to all stakeholders (Table 2) [46].

The obligations and responsibilities of the major professional nephrology societies requires renewed discussion such that advocacy, calls to action and relief efforts are equitable, well-coordinated, aligned and synergistic. Supporting the local nephrology societies when disasters occur is a respectful and likely impactful strategy to maximize effectiveness of kidney care relief. Collectively, societies should advocate with international organizations, governments and industry to enhance collaboration and commitments to contribute.

#### Ethics obligations of the broader stakeholder network

Ethics obligations should extend to all stakeholders positioned to save lives. As such, industry partners such as dialysis and pharmaceutical companies, also have a duty to rescue. If they are not willing to collaborate, all preparedness plans and duties to care become moot. The decision to get involved should not be discretionary or governed by political or economic arguments. Companies should be held accountable for their (un)willingness to assist, provide transparent information on pricing, and support relief efforts in a timely manner by facilitating communication with key decision makers in their organizations. The principles of proportionality, or at least reciprocity, also apply, especially in countries where dialysis has been provided for years before the crisis, supported by the health system, and which have a long history of ‘doing business’ with these companies. Some discussion occurred around the ethics obligations of the pharmaceutical industry regarding global access to COVID-19 vaccines and therapeutics [58]. These arguments should be extended to other lifesaving health products including dialysis supplies and transplant medications.

Ethics obligations should also extend to the media to report transparently and truthfully, to support communication and dissemination of accurate information and to continue to highlight regions in crisis after the acute period [46].

### The obligation to strengthen disaster preparedness

The pandemic has highlighted the ethical imperatives of preparedness and health systems resilience in emergencies, and especially that the needs of people living with non-communicable diseases, such as those with kidney disease, be included in disaster planning [7, 43, 59–61]. Preparedness should include ensuring equity in access to interventions and protections, building technical and logistical capacity, and supporting knowledge and training—all of which are needed to build and maintain trust and accountability, to enhance efficiency and effectiveness of responses, and are crucial for resilient dialysis services (Fig. 1) [2, 62]. Continuing medical education and planning for remote support through telemedicine can optimize responsiveness in emergencies [35]. Transparency and accuracy of communication are recurrent challenges. Preparedness should include establishing clear lines of communication and command in case of crisis [35]. Pre-emptively addressing the social determinants of health, e.g. poverty, education and discrimination, and implementing strategies to support the most vulnerable will not only result in higher standards of health in normal conditions, but will also contribute to greater resilience of individuals in a crisis [63]. Critical to all disaster preparedness, beyond strengthening emergency responses, is mitigation of needs. In the case of kidney disease this calls for strong advocacy to raise awareness among policy makers and communities that kidney disease is preventable and treatable if diagnosed early. A focus on upstream drivers of the kidney disease burden is therefore key to reduce the need for dialysis and transplantation overall.

### Research in emergencies

To understand how best to respond to and meet the needs of people with kidney disease in humanitarian crises it is important to collect data, monitor and evaluate outcomes, and possibly test new strategies [64]. Doing research under emergency conditions is challenging and controversial given the heightened vulnerability of the population, the risk of therapeutic misconception, the challenges in obtaining informed consent and the at times relatively unproven effects of novel interventions being tested, as well as potential diversion of resources and time from the relief activities [65, 66]. It is however becoming increasingly clear that humanitarian action is not always beneficial and may impose harms, and therefore monitoring and evaluation of interventions is important to inform future activities [67]. Indeed, data collection during the Marmara Earthquake in 1999 [68] has provided important insights into the management of crush injury and how to improve preparedness for earthquakes and other disasters, and has created a precedent for data collection in other crises [39, 69–71].

Given the heightened vulnerability of potential research subjects and the instability of the context, the ethical implications of research in emergencies must be carefully considered [66]. According to the World Health Organization, there is an ethical imperative for research to be conducted during public health emergencies [65]. As such, the scientific validity and social value of a study or data collection must be justifiable in the specific context and not impede the emergency response. Careful assessment of risks and benefits must be conducted in collaboration with all stakeholders, participation must be voluntary and well informed, efforts must be made to engage meaningfully with affected communities, and participants should be fairly selected to avoid systematic discrimination, e.g. against pregnant women and children [72]. All research must be subjected to independent ethics review, which can be expedited if standardized template protocols are pre-reviewed before an emergency [66, 73, 74]. Privacy and confidentiality of participants and data must be strictly observed [75]. Data ownership and data sharing must be transparent, fair and respectful, and post-trial access to successful interventions should be assured for the researched population. Risk management strategies for research in emergencies also require additional consideration given many potential unanticipated developments [66].

## CONCLUSIONS

The global kidney community has accumulated important experience in supporting care in diverse disasters around the world. Lessons learned from these disasters have been shared to help optimize local and international responsiveness in emergencies and have highlighted the ethics challenges experienced and the duties and obligations of all stakeholders [2, 52, 76, 77]. Emergencies in fragile settings highlight extreme vulnerability especially of those living on dialysis, but also the broader the systemic neglect of kidney care on the global health agenda [59]. It is unlikely that relief efforts can provide resilient and sustainable dialysis services where these were weak to barely existent prior to a crisis. Emergencies therefore serve as opportunities to advocate for better kidney care at baseline. Solidarity is required across the global kidney community to contribute to development of innovative solutions to provide equitable and affordable kidney care, which includes prioritization of testing and early treatment of CKD to prevent kidney failure. Common sense, and previous experience, indicate that the most successful interventions for humanitarian emergencies were delivered in regions with relatively strong and resilient health systems, and which were able to rapidly scale up dialysis services on site and/or evacuate patients with kidney diseases within the country itself [2]. Strengthening baseline services under non-crisis conditions is therefore a key ethical imperative to enhance resilience during disasters.

## Data Availability

No new data were generated or analysed in support of this research.
